# Time series forecasting using singular spectrum analysis, fuzzy systems and neural networks

**DOI:** 10.1016/j.mex.2020.101015

**Published:** 2020-07-29

**Authors:** Winita Sulandari, S. Subanar, Muhammad Hisyam Lee, Paulo Canas Rodrigues

**Affiliations:** a Study Program of Statistics, Universitas Sebelas Maret, Indonesia; b Departement of Mathematics, Universitas Gadjah Mada, Indonesia; c Department of Mathematical Sciences, Universiti Teknologi Malaysia, Malaysia; d Department of Statistics, Federal University of Bahia, Brazil

**Keywords:** Hybrid methodology, Deterministic model, Nonlinear stochastic model, Weighted fuzzy time series

## Abstract

Hybrid methodologies have become popular in many fields of research as they allow researchers to explore various methods, understand their strengths and weaknesses and combine them into new frameworks. Thus, the combination of different methods into a hybrid methodology allows to overcome the shortcomings of each singular method. This paper presents the methodology for two hybrid methods that can be used for time series forecasting. The first combines singular spectrum analysis with linear recurrent formula (SSA-LRF) and neural networks (NN), while the second combines the SSA-LRF and weighted fuzzy time series (WFTS). Some of the highlights of these proposed methodologies are:

• The two hybrid methods proposed here are applicable to load data series and other time series data.
• The two hybrid methods handle the deterministic and the nonlinear stochastic pattern in the data.
• The two hybrid methods show a significant improvement to the single methods used separately and to other hybrid methods.

## Ghrapical abstract

**Figure:**
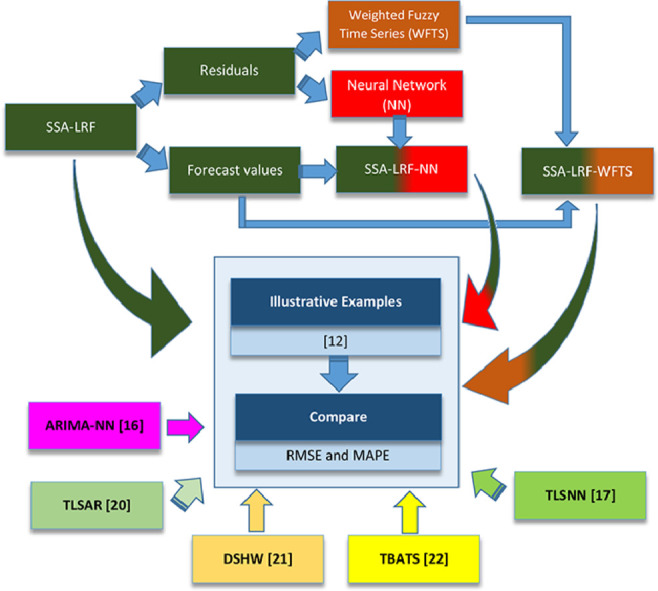

Specifications table

**Table:** 

Subject Area	Energy
More specific subject area	*Load forecasting*
Method name	*SSA-LRF-NN and SSA-LRF-WFTS*
Name and reference of original method	*Sulandari, W., Subanar, Lee, M.H. and Rodrigues, P.C. (2019). Indonesian Electricity Load Forecasting Using Singular Spectrum Analysis, Fuzzy Systems and Neural Networks. Energy.*
Resource availability	

## Method details

The SSA-LRF is a widely used non-parametric method for time series forecasting [1], [2], [3], which is considered as a deterministic model that can be represented by the sum of product of exponential, polynomials and harmonics components [4]. SSA-LRF does not accommodate the random (noise) behavior in the series as its aim is to separate the signal from the noise components. When the noise shows nonlinear relationships, other nonlinear models, such as NN and fuzzy systems can be implemented. In this proposal, the NN and fuzzy systems are used, in combination with the SSA-LRF, to account for the stochastic nonlinearity in the data that cannot be handled by SSA-LRF.

The R package RSSA (http://cran.r-project.org/web/packages/Rssa) can be used to estimate the parameters and calculating the SSA-LRF forecast values. The features of RSSA implementation have been discussed in detail by [5], while further methodological details about SSA can be found in[4,[6], [7]. After fitting the SSA-LRF and obtaining the residuals of the time series, those residuals are then modeled by WFTS or NN.

There are four types of WFTS considered in this hybrid methodology, i.e., Chen's, Yu's, Cheng's, and Lee's method. The procedure of Chen's method was discussed detail in [8]. Yu [9] discussed the WFTS and how it is able to take into account the recurrence and weighting in fuzzy relationship. They consider the weight of the fuzzy relationship as a set of chronological linear functions. Cheng et al. [10] developed an adaptive expectation model where the forecast value at time *t* is calculated by adding the observation value at time *t*−1 with the recent weighted forecasting error (the difference between the initial forecasting value and the observation value at time *t*−1 multiplied by the weighted parameter). Lee's procedure [11] was developed based on Yu's algorithm by setting the weight of fuzzy relationship as an exponential growth function. The source code for these four procedures can be found in the supplementary material.

On the other hand, the NN model can be obtained by using, e.g., the ntstool package available in Matlab. The nonlinear autoregressive were considered with the tansig and purelin functions for the hidden and the output layers, respectively. The model parameters are determined based on the training data by using the Levenberg-Marquardt backpropagation algorithm.

**Fig. 1:**
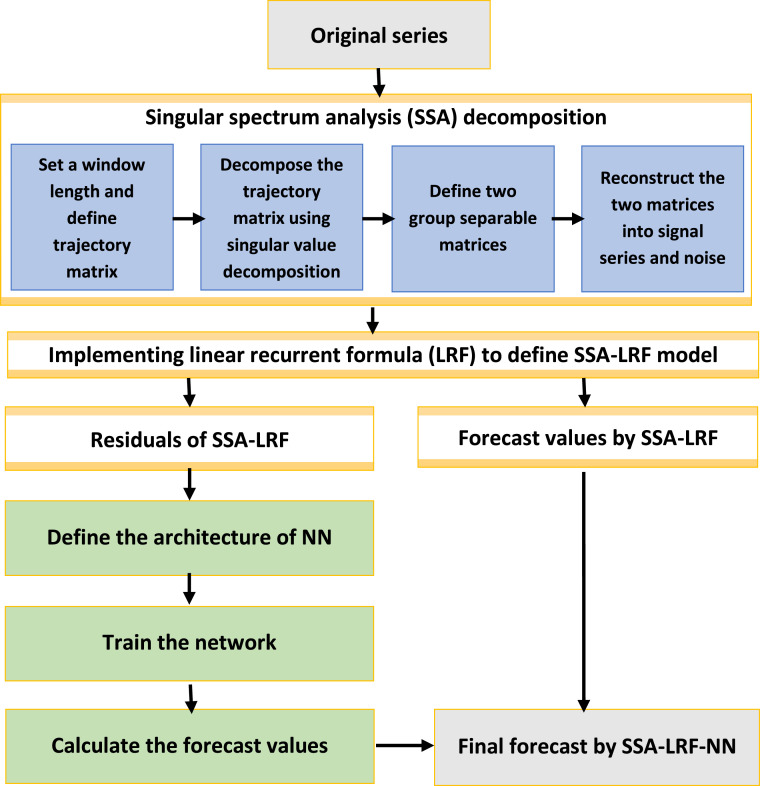
Procedure of the hybrid SSA-LRF-NN method.

The steps of SSA-LRF-NN algorithm presented in this paper are shown in Fig. 1 and the detailes for each step are described as follows ([12]):

**Step 1:** Implementation of the SSA-LRF algorithm to model fit and model forecast the time series data
  **Sub-step 1.1:** Specify the window length, and map the original time series into a trajectory matrix based on the chosen window length. The window length should be close to the middle of the time series and proportional to the seasonal period (e.g., proportional to 12 for monthly data).
  **Sub-step 1.2:** Apply the singular value decomposition (SVD) to decompose the trajectory matrix.
  **Sub-step 1.3:** Define the two separable groups of matrices, one associated to the signal and the other associated to the noise. The use of the w-correlation matrix can be very helpful in this stage.
  **Sub-step 1.4:** Reconstruct the two matrices using the diagonal averaging algorithm, into the signal series and the noise time series.
  **Sub-step 1.5:** Calculate the forecast values for the signal using the linear recurrent formula.
**Step 2:** Combination of SSA-LRF and NN
  **Sub-step 2.1:** Obtain the residuals of the SSA-LRF as described in Step 1.
  **Sub-step 2.2:** Design the architecture of the network by determine the number of input units, the number of nodes in the hidden layer, and specify the activation functions used in the hidden and output layer. For the univariate time series forecasting, we considered one output unit. Meanwhile, the selection of the activation function in the hidden layer can be adjusted according to the range of the data. In this case, we modeled the residuals obtained from SSA-LRF model, which fluctuate around zero. Since the data has negative and positive values, the tansig function is the more appropriate for the hidden layer and the purelin function for the output layer.
  **Sub-step 2.3:** Obtain the weights connecting each input node to each hidden node, and those connecting each hidden node to each output node, using the Levenberg-Marquardt based back-propagation algorithm.
  **Sub-step 2.4:** Calculate the forecast values for the residual of the SSA-LRF using the NN model obtained in Sub-step 2.3.
  **Sub-step 2.5:** Calculate the final forecast values by adding the forecast values obtained by the SSA-LRF (Sub-step 1.5) and the forecast values obtained by the NN (Sub-step 2.4).

The fuzzy time series model is represented as the fuzzy relationships among observations [13], [14], [15]. A fuzzy time series *F*(*t*) can be considered as a linguistic variable on a time series *Y*(*t*), where *t*  ∈  *T*, is a set of time points. In this case, *F*(*t*) is a collection of some fuzzy sets $\mu_{i}\left(t\right)\left(i=1,2,\ldots \right)$, which are regarded as the possible linguistic values of *F*(*t*) defined on *Y*(*t*), while *Y*(*t*) is the universe of discourse on which $\mu_{i}\left(t\right)\left(i=1,2,\ldots \right)$ are defined. The relation between F(t−1) and *F*(*t*) can be written as *F*(*t*-1)→*F*(*t*) if F(t−1) affects *F*(*t*). This kind of fuzzy time series is named first order fuzzy time series and the relation is called fuzzy logical relationship (FLR). Let the linguistic value of F(t−1) be *A_i_* and the linguistic value of *F*(*t*) be *A_j_*, where *A_i_* and *A_j_* are the fuzzy set for the observation at the time t−1 and at the time *t*, respectively. The FLR between the two fuzzy sets can be denoted by *A_i_*→*A_j_* [13], [14], [15].

**Fig. 2:**
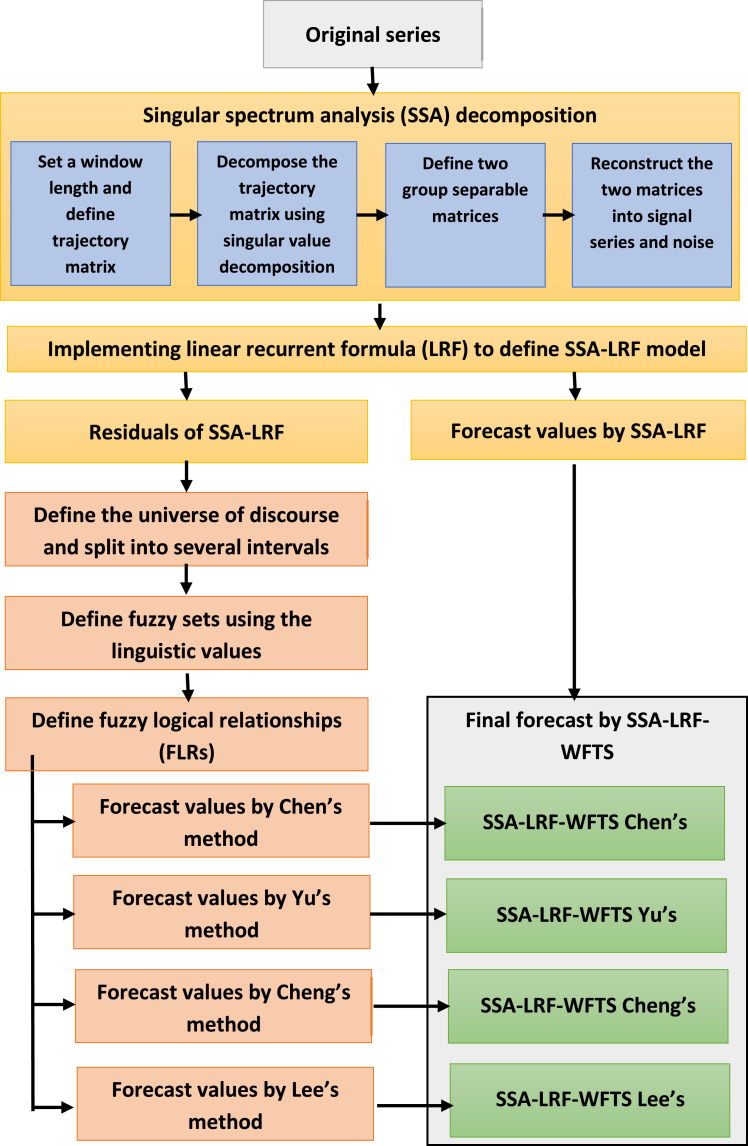
Procedure of the hybrid SSA-LRF-WFTS method.

The steps of SSA-LRF-Fuzzy model presented in this paper are shown in Fig. 2 and the detailes for each step are described as follows ([12]):

**Step 1:** Analog to the first step of SSA-LRF-NN procedure.
**Step 2:** Combination of the SSA-LRF and the fuzzy time series
  **Sub-step 2.1:** Obtain the residuals of the SSA-LRF as described in Step 1.
  **Sub-step 2.2:** Set the universe of discourse, *U*, and split it into several equal length intervals.
    Let *D_min_* and *D_max_* be the minimum and maximum residual defined in Sub-step 2.1, respectively. The universe of discourse, *U*, can be define as $U\;=\;[D_{min}\;-\;D_{1},\;D_{max}+\;D_{2}]$ where *D*_1_ and *D*_2_ are the proper positive numbers. In the case that *U* is partitioned into *n* equal intervals $u_{1},u_{2},\;\ldots ,\;u_{n}$, the length of the interval, *l,* can be defined as $l=[\left(D_{max}+D_{2}\right)-\left(D_{min}-D_{1}\right)/n$.
  **Sub-step 2.3:** Define the fuzzy sets $A_{1},\;A_{2},\;\ldots ,\;A_{n}$, as the linguistic values of the linguistic variable on the observed time series. Each $A_{i},\;i\;=\;1,\;\ldots ,\;n,$ is defined by the intervals found in Sub-step 2.2 [13], [14], [15], and can be written as [8],
    $$A_{i}\;=\ldots +0/u_{i-2}+0.5/u_{i-1}+1/u_{i}+0.5/u_{i+1}+0/u_{i+2}+...$$
    where the maximum membership value of *A_i_* occurs in the interval *u_i_*.
  **Sub-step 2.4:** Fuzzify the residuals obtained in Sub-step 2.1 by considering the fuzzy sets as defined in Sub-step 2.3.
  **Sub-step 2.5**: Obtain the fuzzy logical relationships (FLRs) according to
    (1) Chen's method [8]
    (2) Yu's method [9]
    (3) Cheng's method [10]
    (4) Lee's method [11]
  **Sub-step 2.6**: Calculate the forecast values for the residuals based on FLRs according to the related method as determined in Sub-step 2.5.
  **Sub-step 2.7**: Calculate the final forecast values by adding the forecast values obtained by the SSA-LRF (Step 1) and the forecast values obtained by the fuzzy model (Sub-step 2.6).

Further details about the results of the application to electricity load forecasting that validate the usefulness of the proposed methods can be found in [12]. In [12], all the results obtained from the two proposed hybrid methodologies, SSA-LRF-NN and SSA-LRF-WFTS, were compared with the results from the standard SSA-LRF framework. In order to provide more objective evaluation to the accuracy performance of the forecasting results using the two proposed methods, we provide comparisons with standard methods for time series forecasting and other two hybrid methods, the ARIMA-NN [16] and the TLSNN (two levels seasonal neural network) model [17]. In the ARIMA-NN model [16], ARIMA was proposed to handle the linear relationship in the data, while the NN captures the nonlinearity pattern in the data. In this study, the model parameters of the ARIMA model were estimated with the auto.arima function of the R package forecast [18], while the parameters of NN are estimated by nnetar function from the same R package. As stated in [19], NN do not only work well for handling the nonlinearity relationship in the data, but also linear relationships in the time series data. Recently, [17] proposed the TLSNN model, which consists of two parts to estimate the deterministic and the stochastic components. The deterministic component, including the trend and the oscillatory component, is estimated by the SSA, while the stochastic component, including the residuals of the deterministic model, is modeled by a NN.

The other methods that were used for comparison in this paper are: the two-level seasonal autoregressive (TLSAR) model [20], the double seasonal Holt-Winter (DSHW) model [21], and the TBATS (Trigonometric, Box-Cox transform, ARMA errors, Trend, and Seasonal components) model [22]. The success of TLSAR, TBATS, DSHW, and TLSNN in modeling the load electricity time series in Indonesia can be seen in [12,23]. The results for the comparisons, based on the root mean square error (RMSE) and on the mean absolute percentage error (MAPE), between the two proposed hybrid algorithms and the competing models can be seen in Table 1, Table 2, Table 3, Table 4, Table 5. Further details can be found in [12].

**Table 1:** RMSEs and MAPEs for all methods considering the training and the testing data of the Java-Bali load series in 2014.

Method	RMSE		MAPE (%)	
	Training	Testing	Training	Testing
SSA-LRF	816.94	764.50	3.23	2.88
SSA-LRF-WFTS Chen's	501.22	512.05	1.97	1.90
SSA-LRF-WFTS Yu's	503.27	508.02	1.98	1.92
SSA-LRF-WFTS Cheng's	504.50	502.79	1.94	1.85
SSA-LRF-WFTS Lee's	507.32	522.27	2.00	1.98
SSA-LRF-NN (24-9-1)	149.87	161.66	0.57	0.64
TLSAR*	396.34	430.75	1.53	1.74
TBATS	241.26	318.12	0.85	1.48
DSHW	206.37	1013.01	0.74	4.14
ARIMA(5,0,4)-NN(31-16-1)	94.06	1545.00	0.38	6.76
TLSNN(29-15-1)	101.61	273.46	0.36	1.04

⁎ period: 168, number of harmonics: 21, ARIMA(4,1,3).

**Table 2:** RMSEs and MAPEs for all methods considering the training and the testing data of the Java-Bali load series in 2015.

Method	RMSE		MAPE (%)	
	Training	Testing	Training	Testing
SSA-LRF	473.25	379.31	3.23	1.49
SSA-LRF=WFTS Chen's	385.96	334.46	1.47	1.30
SSA-LRF=WFTS Yu's	386.67	335.54	1.47	1.30
SSA-LRF=WFTS Cheng's	388.28	346.90	1.45	1.34
SSA-LRF=WFTS Lee's	391.77	354.15	1.48	1.37
SSA-LRF-NN (18-10-1)	155.68	142.82	0.59	0.56
TLSAR*	398.79	408.76	1.48	1.55
TBATS	239.22	206.02	0.82	0.85
DSHW	162.31	159.28	0.53	0.59
ARIMA(3,0,2)-NN(30-16-1)	85.68	1656.00	0.30	5.95
TLSNN(30-16-1)	83.32	205.93	0.30	0.77

⁎ period: 168, number of harmonics: 21, ARIMA(5,0,3) with zero mean.

**Table 3:** RMSEs and MAPEs for all methods considering the training and the testing data of the Java-Bali load series in 2016.

Method	RMSE		MAPE (%)	
	Training	Testing	Training	Testing
SSA-LRF	703.72	447.30	2.83	1.74
SSA-LRF-WFTS Chen's	247.55	179.44	0.96	0.69
SSA-LRF-WFTS Yu's	255.27	189.57	1.02	0.75
SSA-LRF-WFTS Cheng's	226.94	172.03	0.87	0.63
SSA-LRF-WFTS Lee's	266.94	211.28	1.08	0.81
SSA-LRF- NN 18-10-1	152.71	129.90	0.55	0.50
TLSAR*	343.83	385.98	1.29	1.41
TBATS	225.29	293.72	0.77	0.96
DSHW	151.85	191.00	0.53	0.69
ARIMA(5,0,4)-NN(31,16-1)	81.22	1770.05	0.28	6.61
TLSNN(31-16-1)	93.13	366.06	032	1.43

⁎ period: 168, number of harmonics: 21, ARIMA(4,1,4).

**Table 4:** RMSEs and MAPEs for all methods considering the training and the testing data of Bawen load time series.

Method	RMSE		MAPE (%)	
	Training	Testing	Training	Testing
SSA-LRF	0.39	0.58	2.61	2.86
SSA-LRF-WFTS Chen's	0.30	0.27	0.97	0.66
SSA-LRF-WFTS Yu's	0.30	0.28	1.02	0.78
SSA-LRF-WFTS Cheng's	0.30	0.27	1.00	0.70
SSA-LRF-WFTS Lee's	0.30	0.29	1.10	0.95
SSA-LRF-NN(48-10-1)	0.10	0.10	0.37	0.32
TLSAR*	0.35	0.32	1.12	1.20
TBATS	0.27	0.39	0.79	1.71
DSHW	0.30	0.48	0.68	2.09
ARIMA(2,1,2)-NN(30-16-1)	0.07	0.73	0.19	3.07
TLSNN(20-10-1)	0.18	0.26	0.57	0.86

⁎ period: 336, number of harmonics: 14, ARIMA(3,0,4) with zero mean.

**Table 5:** RMSEs and MAPEs for all methods considering the training and the testing data the weekly US ending stocks of the total gasoline.

Method	RMSE		MAPE (%)	
	Training	Testing	Training	Testing
SSA-LRF	6486.3	5215.7	2.43	1.93
SSA-LRF-WFTS Chen's	2478.9	3315.2	0.87	1.04
SSA-LRF-WFTS Yu's	2299.9	3372.8	0.85	1.05
SSA-LRF-WFTS Cheng's	2281.9	3410.8	0.85	1.05
SSA-LRF-WFTS Lee's	2275.2	3324.8	0.85	1.05
SSA-LRF-NN (9-8-1)	2047.7	2450.1	0.76	0.76
SSA-LRF-NN (10-9-1)	2008.3	2254.0	0.75	0.86
TLSAR*	2282.4	7023.8	0.85	2.85
TBATS	2236.47	5423.4	0.83	2.24
DSHW	-	-	-	-
ARIMA(2,1,2)-NN(1-1-1)	2345.1	7746.0	0.88	2.10
TLSNN(3-2-1)	2239.9	6649.5	0.84	2.73

⁎ period: 52, number of harmonics: 3, ARIMA(0,1,2).

Table 5 does not include the results for the DSHW model because this model, proposed by [21], intends to handle double seasonal patterns in the series and the weekly gasoline data does not show two seasonal patterns. It should be noted that a particular model that provides better performance in one case may not necessarily give the same results in another case. Further, [12] have not taken into account the effect of holidays or special days as in [20].

**Fig. 3:**
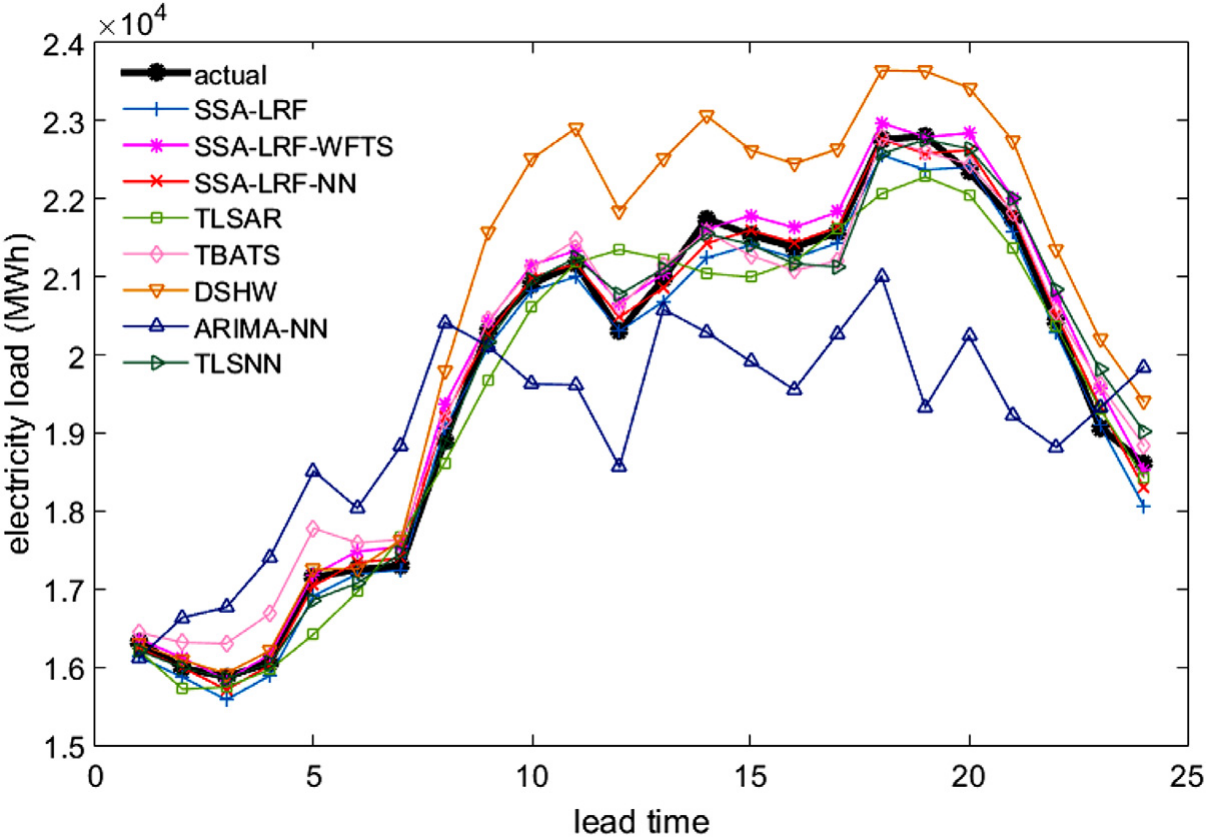
Actual data and forecast values for the different models, considering the test data of the Java-Bali load time series in 2014.

**Fig. 4:**
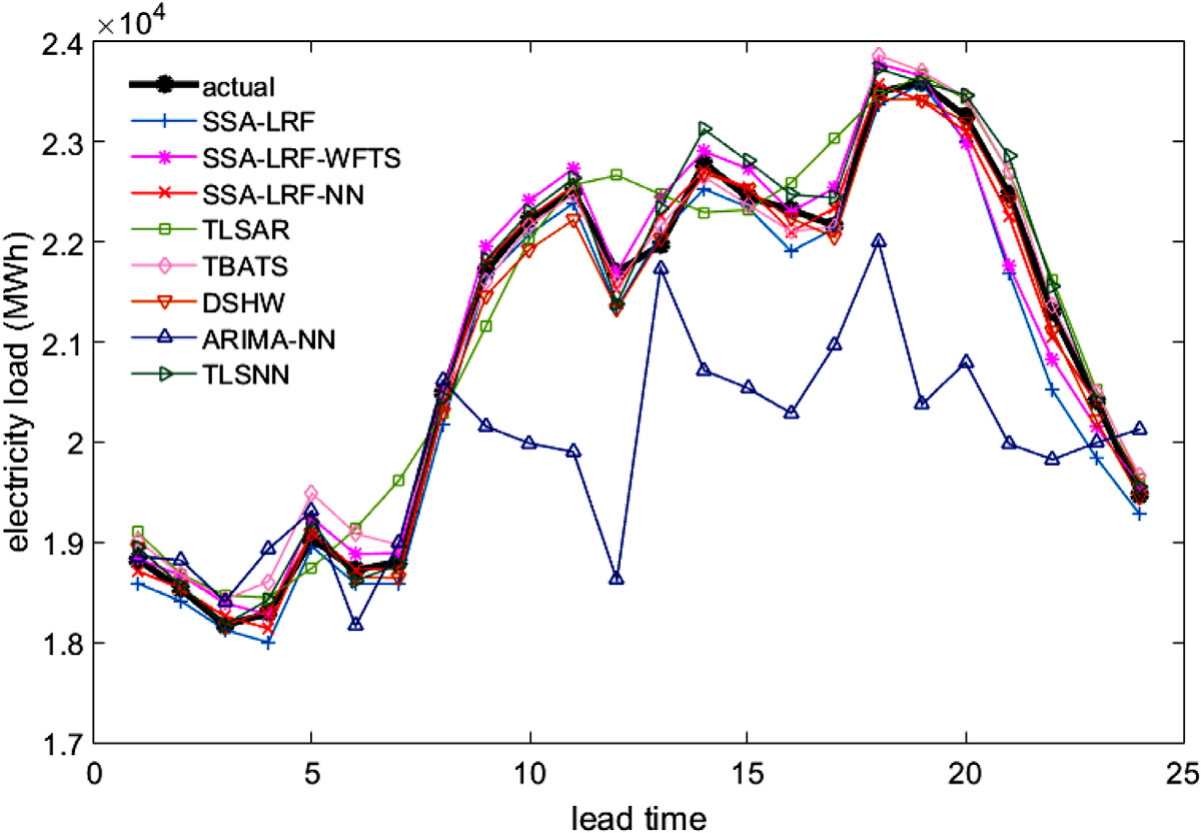
Actual data and forecast values for the different models, considering the test data of the Java-Bali load time series in 2015.

**Fig. 5:**
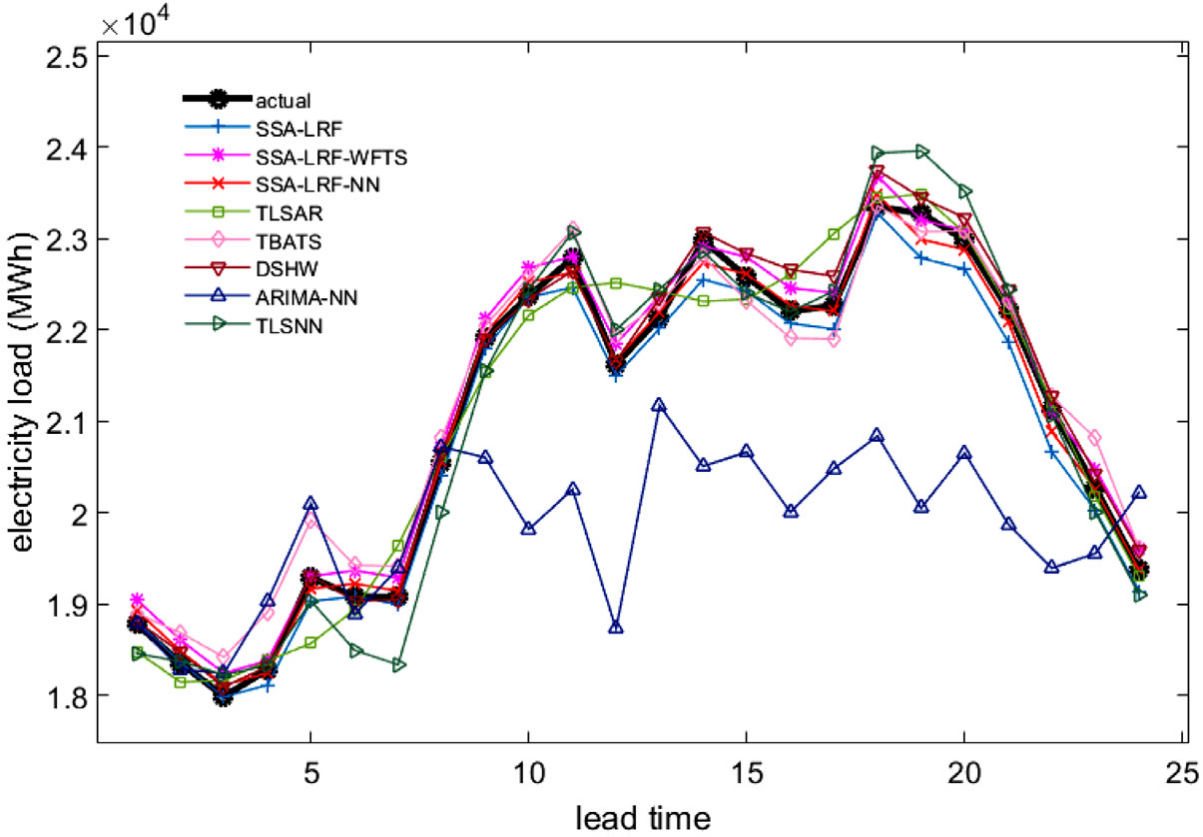
Actual data and forecast values for the different models, considering the test data of the Java-Bali load time series in 2016.

**Fig. 6:**
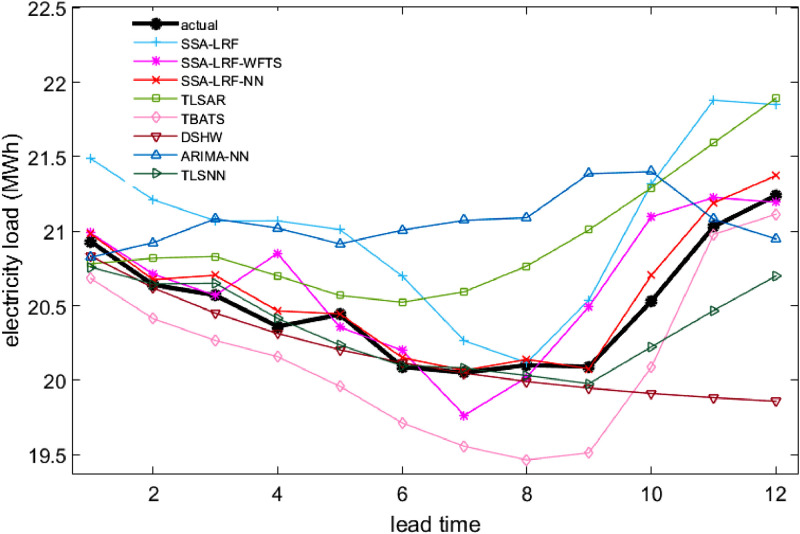
Actual data and forecast values for the different models, considering the test data of the Bawen load time series in 2014.

**Fig. 7:**
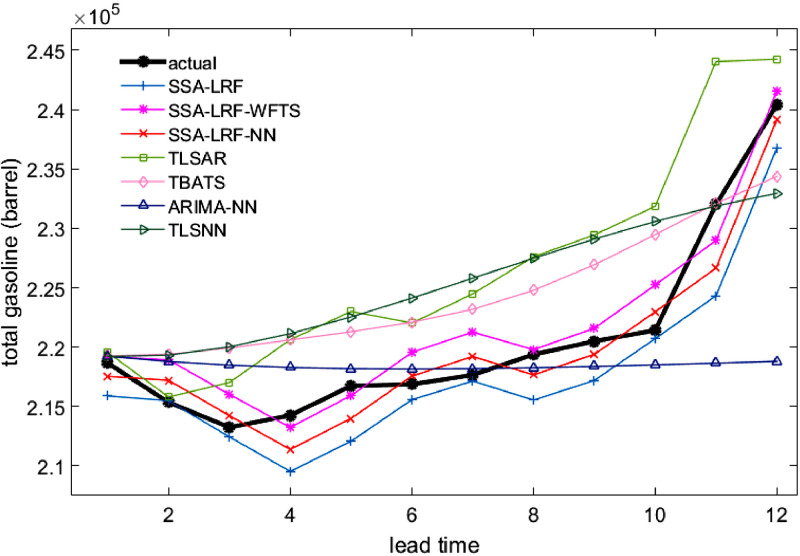
Actual data and forecast values for the different models, considering the test data of the total gasoline.

Comparison plots to show the closeness between the actual and forecast values for the different models, considering all data sets under study, are depicted in Fig. 3, Fig. 4, Fig. 5, Fig. 6, Fig. 7. Moreover, the two-sided Diebold-Mariano test was used to determine whether the forecast values are significantly different from each other are presented in Table 6. We considered a significance level of 0.05, being the p-values above that threshold associated to no significant difference between the forecasts obtained by method A and method B. When the p-values are lower than 0.05, one-side hypothesis tests are conducted to determine which forecast method provides more accurate results. The results for the one-sided Diebold-Mariano tests [37] are also included in Table 6 under the form of superscript close to the p-values. All calculations were done by using the dm.test function from the R package forecast.

**Table 6:** The p-values obtained from the two-sided Diebold-Mariano test between methods for the five time series discussed in this study.

Method		Data				
A	B	1	2	3	4	5
SSA-LRF	SSA-LRF-Fuzzy*	0.0251^b^	0.1452⁎⁎	0.0023^b^	0.0007^b^	0.1227⁎⁎
	SSA-LRF-NN	0.0029^b^	0.0026^b^	0.0028^b^	0.0005^b^	0.0569⁎⁎
	TLSAR	0.0215⁎⁎	0.7328⁎⁎	0.1986⁎⁎	0.5652⁎⁎	0.2881⁎⁎
	TBATS	0.0115⁎⁎	0.0185^b^	0.1172⁎⁎	0.0637⁎⁎	0.8041⁎⁎
	DSHW	0.0283^a^	0.0052^b^	0.0090^b^	0.8733⁎⁎	-
	ARIMA-NN	0.0033^a^	0.0005^a^	0.0001^a^	0.3191⁎⁎	0.4350⁎⁎
	TLSNN	0.0085^b^	0.0100^b^	0.2859⁎⁎	0.0002^b^	0.2566⁎⁎
SSA-LRF-Fuzzy*	SSA-LRF-NN	0.0057⁎⁎	0.0024^b^	0.2086⁎⁎	0.0588⁎⁎	0.4088⁎⁎
	TLSAR	0.3251⁎⁎	0.3580⁎⁎	0.0076^a^	0.0096^a^	0.0191^a^
	TBATS	0.0715⁎⁎	0.0271^b^	0.1383⁎⁎	0.1169⁎⁎	0.0070^a^
	DSHW	0.0003^a^	0.0061^b^	0.6698⁎⁎	0.2342⁎⁎	-
	ARIMA-NN	0.0006^a^	0.0004^a^	0.0000^a^	0.0080^a^	0.1643⁎⁎
	TLSNN	0.0408^b^	0.0167^a^	0.0066^a^	0.8940^a^	0.0013^a^
SSA-LRF-NN	TLSAR	0.0057^a^	0.0088^a^	0.0087^a^	0.0039^a^	0.0083^a^
	TBATS	0.0057^a^	0.0874⁎⁎	0.0457^a^	0.0041^a^	0.0011^a^
	DSHW	0.0000^a^	0.5789⁎⁎	0.1221⁎⁎	0.1228⁎⁎	-
	ARIMA-NN	0.0001^a^	0.0003^a^	0.0000^a^	0.0057^a^	0.1732⁎⁎
	TLSNN	0.0506⁎⁎	0.0166^a^	0.0012^a^	0.0910⁎⁎	0.0004^a^
TLSAR	TBATS	0.1546⁎⁎	0.0329⁎⁎	0.3108⁎⁎	0.0723⁎⁎	0.1770⁎⁎
	DSHW	0.0000^a^	0.0083^b^	0.0257^b^	0.8620⁎⁎	-
	ARIMA-NN	0.0003^a^	0.0005^a^	0.0000^a^	0.0422^a^	0.7936⁎⁎
	TLSNN	0.0590⁎⁎	0.0202⁎⁎	0.7965⁎⁎	0.0163^b^	0.8003⁎⁎
TBATS	DSHW	0.0000^a^	0.2699⁎⁎	0.0989⁎⁎	0.4494⁎⁎	-
	ARIMA-NN	0.0002^a^	0.0004^a^	0.0000^a^	0.0103^a^	0.4640⁎⁎
	TLSNN	0.3632⁎⁎	0.9983⁎⁎	0.1931⁎⁎	0.1922⁎⁎	0.0030^a^
DSHW	ARIMA-NN	0.0203^a^	0.0003^a^	0.0000^a^	0.4425⁎⁎	-
	TLSNN	0.0000^b^	0.1894⁎⁎	0.0093^a^	0.1335⁎⁎	-
ARIMA-NN	TLSNN	0.0002^b^	0.0003^b^	0.0000^b^	0.01862^b^	0.7252⁎⁎

Data 1: hourly Java-Bali load series in 2014.

Data 2: hourly Java-Bali load series in 2015.

Data 3: hourly Java-Bali load series in 2016.

Data 4: half hourly Bawen load series.

Data 5: weekly US ending stocks of the total gasoline.

a means method A is more accurate than method B.

b means method B is more accurate than method A.

⁎ represents the chosen model determined based on the smallest values of MAPE and RMSE in the testing data.

⁎⁎ means method A and method B has no different accuracy.

The results presented in Table 1, Table 2, Table 3, Table 4, Table 5, Table 6 and in Figs. 3–7 give a clear picture on the overall better performance, in terms of forecasting ability, of the two methods presented in this paper, when compared with standard individual and hybrid methods available in the literature. Moreover, since the proposed two hybrid approaches SSA-LRF-NN and SSA-LRF-WFTS outperform the SSA-LRF, they are also expected to outperform other methods that SSA-LRF outperforms, e.g. [23], [24].

The forecasting results of the SSA-LRF-WFTS hybrid model may be further improved by applying the higher order fuzzy time series as discussed in [26], [27], [28]. The performance of the fuzzy model is influenced by the universe of discourse selection, length of interval, FLR, and defuzzification. Related literature can be found in [29], [30], [31]. In the case of data contamination with outlying observations, further improvement related to the SSA part of the model can be obtained by considering a robust SSA algorithm [25,32]. A more parsimonious adaptation of the recurrent forecast algorithm [33] or of the vector forecast algorithm [34] can also be considered to improve the forecasting ability of the SSA part of the model. Based on the M4 competition, the hybrid statistical and machine learning approach produces more accurate forecasts and more precise prediction intervals than the combination of statistical approaches [35]. However, it should be noted that more complex models do not guarantee more accurate forecasts than the simpler models [36]. Therefore, caution should be taken when selecting the forecasting algorithm, depending on the kind of data and parsimony required for the model.

## Declaration of Competing Interest

The authors declare that they have no known competing financial interests or personal relationships that could have appeared to influence the work reported in this paper.

## References

[1] Mahmoudvand R., Konstantinides D., Rodrigues P.C. (2017). Forecasting mortality rate by multivariate singular spectrum analysis. Appl. Stochastic Models Bus. Ind..

[2] Mahmoudvand R., Rodrigues P.C. (2018). A new parsimonious recurrent forecasting model in singular spectrum analysis. J. Forecast..

[3] Mahmoudvand R., Rodrigues P.C., Yarmohammadi M. (2019). Forecasting daily exchange rates: a comparison between SSA and MSSA. RevStat-Stat. J..

[4] Golyandina Nina., Nekrutkin V., Zhigljavsky A. (2001). Analysis of Time Series Structure: SSA and Related Techniques.

[5] Golyandina N., Korobeynikov A. (2014). Basic Singular Spectrum Analysis and forecasting with R. Computat. Stat. Data Anal..

[6] Golyandina N., Zhigljavsky A. (2013). Singular Spectrum Analysis for Time Series.

[7] Rodrigues P.C., Mahmoudvand R. (2018). The benefits of multivariate singular spectrum analysis over the univariate version. J. Frankl. Inst..

[8] Chen S.-M. (1996). Forecasting enrollments based on fuzzy time series. Fuzzy Sets Syst..

[9] Yu H.K. (2005). Weighted fuzzy time series models for TAIEX forecasting. Physica A.

[10] Cheng C.H., Chen T.L., Teoh H.J., Chiang C.H. (2008). Fuzzy time-series based on adaptive expectation model for TAIEX forecasting. Expert Syst. Appl..

[11] Lee M.H., Suhartono (2012). A weighted fuzzy time series model for forecasting seasonal data. J. Qual. Meas. Anal..

[12] Sulandari W., Subanar M.H.Lee, Rodrigues P.C. (Jan. 2020). Indonesian electricity load forecasting using singular spectrum analysis, fuzzy systems and neural networks. Energy.

[13] Song Q., Chissom B.S. (1993). Forecasting enrollments with fuzzy time series – part I. Fuzzy Sets Syst..

[14] Song Q., Chissom B.S. (1993). Fuzzy time series and its models. Fuzzy Sets Syst..

[15] Song Q., Chissom B.S. (1994). Forecasting enrollments with fuzzy time series – part II. Fuzzy sets and systems.

[16] Zhang G.P. (2003). Time series forecasting using a hybrid ARIMA and neural network model. Neurocomputing.

[17] Sulandari W., Subanar S., Suhartono S., Utami H., Lee M.H., Rodrigues P.C. (2020). SSA-based hybrid forecasting models and applications. Bull. Electr. Eng. Inform..

[18] Makridakis S., Hyndman R.J., Petropoulos F. (2020). Forecasting in social settings: the state of the art. Int. J. Forecast..

[19] Zhang G., Patuwo B.E., Hu M.Y. (1998). Forecasting with artificial neural networks: the state of the art. Int. J. Forecast..

[20] Soares L.J., Medeiros M.C. (2008). Modeling and forecasting short-term electricity load: A comparison of methods with an application to Brazilian data. International J. Forecast..

[21] Taylor J.W. (2003). Short-term electricity demand forecasting using double seasonal exponential smoothing. J. Oper. Res. Soc..

[22] De Livera A.M., Hyndman R.J., Snyder R.D. (2011). Forecasting time series with complex seasonal patterns using exponential smoothing. J. Am. Stat. Assoc..

[23] Sulandari W., Subanar S., Suhartono S., Utami H. (2016). Forecasting electricity load demand using hybrid exponential smoothing-artificial neural network model. Int. J. Adv. Intell. Inform..

[24] Hassani H., Heravi S., Zhigljavsky A. (2009). Forecasting European industrial production with singular spectrum analysis. Int. J. Forecast..

[25] Rodrigues P.C., Pimentel J., Messala P., Kazemi M. (2020). The decomposition and forecasting of mutual investment funds using singular spectrum analysis. Entropy.

[26] Chen S.-M. (2002). Forecasting enrollments based on high-order fuzzy time series. Cybern. Syst..

[27] Chen M.-Y. (2014). A high-order fuzzy time series forecasting model for internet stock trading. Future Gener. Comput. Syst..

[28] Aladag C.H., Egrioglu E., Yolcu U., Uslu V.R. (2014). A high order seasonal fuzzy time series model and application to international tourism demand of Turkey. J. Intell. Fuzzy Syst..

[29] Singh P. (2017). High-order fuzzy-neuro-entropy integration-based expert system for time series forecasting. Neural Comput. Appl..

[30] Singh P., Dhiman G. (2018). A hybrid fuzzy time series forecasting model based on granular computing and bio-inspired optimization approaches. J. Comput. Sci..

[31] Singh P. (2020). A novel hybrid time series forecasting model based on neutrosophic-PSO approach. Int. J. Mach. Learn. Cybern..

[32] Rodrigues P.C., Lourenço V., Mahmoudvand R. (2018). A robust approach to singular spectrum analysis. Qual. Reliab. Eng. Int..

[33] Mahmoudvand R., Rodrigues P.C. (2018). A new parsimonious recurrent forecasting model in singular spectrum analysis. J. Forecast..

[34] Rodrigues P.C., Mahmoudvand R. (2020). A new approach for the vector forecast algorithm in singular spectrum analysis. Commun. Stat..

[35] Makridakis S., Spiliotis E., Assimakopoulos V. (Oct. 2018). The M4 Competition: results, findings, conclusion and way forward. Int. J. Forecast..

[36] Makridakis S., Hibon M. (2000). The M3-Competition: results, conclusions and implications. Int. J. Forecast..

[37] Diebold F.X., Mariano R.S. (1995). Comparing predictive accuracy. Journal of Business & Economic Statistics.

